# Supplementary material for: Coherent ultrafast spin-dynamics probed in three dimensional topological insulators
Source: Sci Rep. 2015 Oct 29;5:15304. doi: 10.1038/srep15304 (PMC4625143; doi:10.1038/srep15304)
Supplement: Supplementary Information [file srep15304-s1.pdf]

Supplementary Information

Coherent ultrafast spin-dynamics probed in three dimensional topological insulators

[1,2]F. Boschini, [1]M. Mansurova, [3]G. Mussler, [3]J. Kampmeier, [3]D. Grützmacher, [4]L. Braun, [5]F. Katmis, [5]J. S. Moodera, [6]C. Dallera, [6]E. Carpene, [7]C. Franz, [7]M. Czerner, [7]C. Heiliger, [4]T. Kampfrath, [8]M. Münzenberg

1) I. Physikalisches Institut, Georg-August-Universität Göttingen, Friedrich-Hund-Platz 1, 37077 Göttingen, Germany, 2) Dipartimento di Fisica, Politecnico di Milano, 20133 Milan, Italy, 3) Peter Grünberg Institut (PGI-9) and Jülich-Aachen Research Alliance (JARA-FIT), Forschungszentrum Jülich, 52425 Jülich, Germany, 4) Department of Physical Chemistry, Fritz Haber Institute, Faradayweg 4-6, 14195 Berlin, Germany, 5) Massachusetts Institute of Technology, Cambridge, Massachusetts 02139, USA, 6) IFN-CNR, Dipartimento di Fisica, Politecnico di Milano, 20133 Milan, Italy, 7) I. Physikalisches Institut, Justus-Liebig-Universität Gießen, 35392 Gießen, Germany, 8) Institut für Physik, Ernst-Moritz-Arndt Universität Greifswald, Felix-Hausdorff-Straße 6, 17489 Greifswald, Germany

1. Element resolved band structure

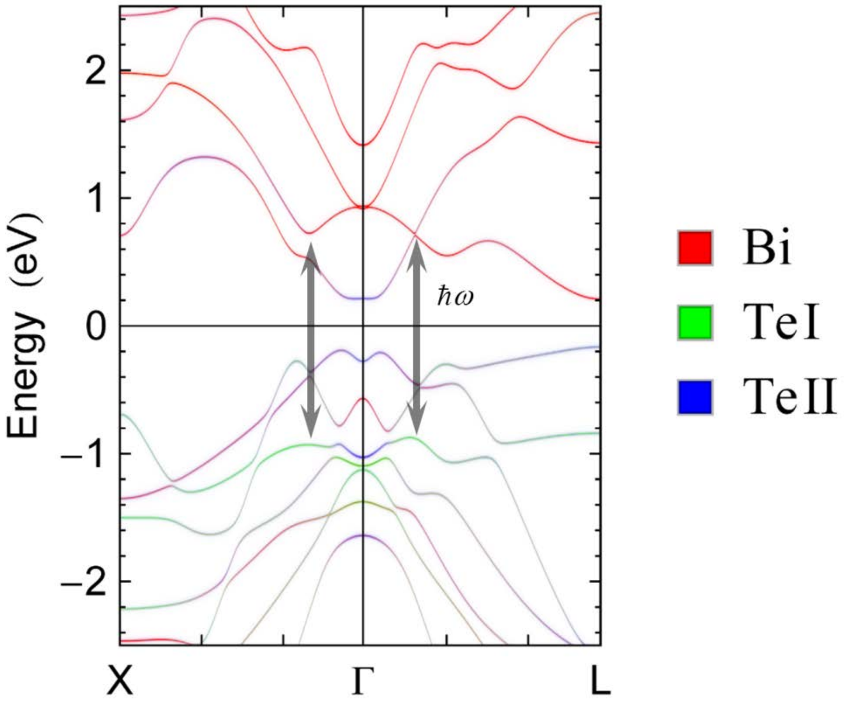

Fig. 1 Calculated band structure: showing Bi and Te character of the states that can be related to the optical transitions for the wavelength λ= 800 nm (photon energy 1.55 eV).

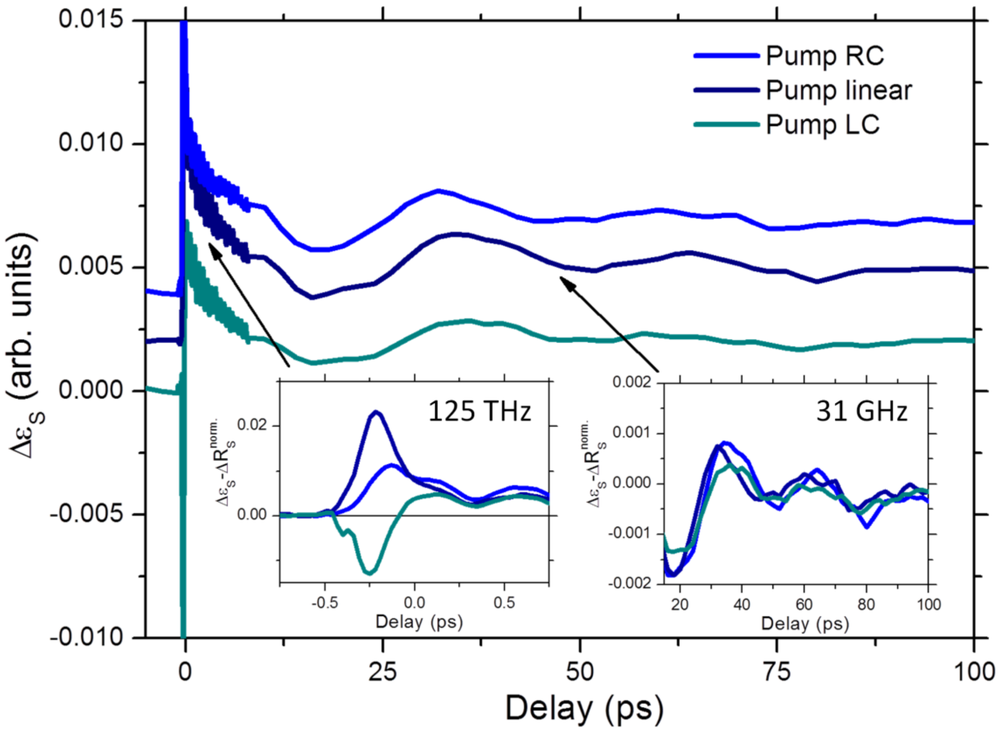

Fig. 2 Time resolved birefringence a) Dynamics on short and long time scales for left circularly (LC), linearly and right circularly (RC) polarized pump pulses. Inset left: polarization dependence on the ultrafast time scale. Inset right: the dynamic mode at 31 GHz shows no dependence on the pump-pulse's handedness.
